# Exploring the Most Promising Stem Cell Therapy in Liver Failure: A Systematic Review

**DOI:** 10.1155/2019/2782548

**Published:** 2019-12-01

**Authors:** Jeanne AdiwinataPawitan

**Affiliations:** ^1^Department of Histology, Faculty of Medicine Universitas Indonesia, Jakarta, Indonesia; ^2^Stem Cell Medical Technology Integrated Service Unit, Dr.CiptoMangunkusumo General Hospital/Faculty of Medicine Universitas Indonesia, Jakarta, Indonesia; ^3^Stem Cell and Tissue Engineering Research Center, Indonesia Medical Education and Research Institute (IMERI), Faculty of Medicine Universitas Indonesia, Jakarta, Indonesia

## Abstract

**Background:**

Alternative approaches to transplantation for liver failure are needed. One of the alternative approaches is stem cell therapy. However, stem cell therapy in liver failure is not standardized yet, as every centre have their own methods. This systematic review is aimed at compiling and analyzing the various studies that use stem cells to treat liver failure, to get an insight into potential protocols in terms of safety and efficacy by comparing them to controls.

**Methods:**

This systematic review was done according to PRISMA guidelines and submitted for registration in PROSPERO (registration number CRD42018106119). All published studies in PubMed/MEDLINE and Cochrane Library, using key words: “human” and “stem cell” AND “liver failure” on 16^th^ June 2018, without time restriction. In addition, relevant articles that are found during full-text search were added. Inclusion criteria included all original articles on stem cell use in humans with liver failure. Data collected included study type, treatment and control number, severity of disease, concomitant therapy, type and source of cells, passage of cells, dose, administration route, repeats, and interval between repeats, outcomes, and adverse events compared to controls. Data were analyzed descriptively to determine the possible causes of adverse reactions, and which protocols gave a satisfactory outcome, in terms of safety and efficacy.

**Results:**

There were 25 original articles, i.e., eight case studies and 17 studies with controls.

**Conclusion:**

Among the various adult stem cells that were used in human studies, MSCs from the bone marrow or umbilical cord performed better compared to other types of adult stem cells, though no study showed a complete and sustainable performance in the outcome measures. Intravenous (IV) route was equal to invasive route. Fresh or cryopreserved, and autologous or allogeneic MSCs were equally beneficial; and giving too many cells via intraportal or the hepatic artery might be counterproductive.

## 1. Introduction

The gold standard therapy for liver failure is liver transplant. However, there are discrepancies between liver supply and demand for transplantation. In the USA, Habka et al. stated that from 16,000 patients who were registered to receive liver transplantation, only 38% could get the transplant [1]. Moreover, the number of patients who need a liver transplant is on the rise each year, which in 2016 the rise in demand was 10% compared to the demand in 2015 [2]. Limited numbers of donors and transplant rejections are problems that liver failure patients have to face. A study on adult living donor transplantation showed that the hazard ratio for chronic and acute rejection was 6.93 (*p* = 0.006) and 2.96 (*p* = 0.017), respectively [3]. Attempts to increase the potential of altruistic organ donation through social media is promising, but not without problems, as there is a possibility that a potential living donor may become a victim of inappropriate social media campaign [4].

Therefore, alternative approaches other than transplantation are needed. One of the alternative approaches is stem cell therapy. However, stem cell therapy in liver failure is not standardized yet, as every centre have their own methods, in terms of the type of cells, the dose, route, and outcome measures to assess the success of therapy. This systematic review is aimed at compiling and analyzing the various studies that use adult stem cells to treat liver failure, to get an insight into potential protocols in terms of safety and efficacy by comparing them to controls.

## 2. Materials and Methods

This systematic review was done according to preferred reporting items for systematic review (PRISMA) guidelines and submitted for registration in PROSPERO (registration number CRD42018106119). All published studies in PubMed/MEDLINE and Cochrane Library, using key words: “human” and “stem cell” AND “liver failure” on 16^th^ June 2018, without time restriction. In addition, relevant articles that are found during full-text search were added.

### 2.1. Inclusion Criteria

All original articles on adult stem cell use in humans with liver failure were included. Exclusion criteria included articles in the non-English literature such as Chinese and German language, and studies on stem cell therapy in liver failure that did not assess both the adverse events and any of common liver failure study outcomes, i.e., liver failure symptoms and signs (ascites, lower limb edema, and jaundice), lab findings (albumin, alanine aminotransferase (ALT), aspartate aminotransferase (AST), total bilirubin, direct bilirubin, prothrombin time and concentration), or liver failure complications (hematemesis, melena, encephalopathy). Also studies that gave granulocyte-macrophage colony-stimulating factor (GM-CSF) only without stem cells were excluded.

Data collected includes type of study, number of participant, number of treatments and controls, severity of disease, concomitant therapy besides of stem cell therapy, type and source of cells, passage of cells, dose, vehicle type, vehicle amount, administration route, repeats, and interval between repeats, outcomes, and adverse events compared to controls.

### 2.2. Data Analysis

The studies were grouped and tabulated according to types of studies, which were divided into case studies (studies without control) and controlled clinical trials. Further, the number of cases, and outcome-related variables, i.e., type and source of cells, passage, dose, combination with other therapies and outcome were tabulated. In addition, stem cell therapy adverse reaction-related variables, i.e., cell type and sourc, vehicle type and volume, route of administration, and adverse reaction, were also tabulated.

Data were analyzed descriptively to determine the possible causes of adverse reactions, and which protocols gave a satisfactory outcome, in terms of safety and efficacy. Further animal studies and pluripotent cell-derived cell therapy were discussed.

## 3. Results and Discussion

### 3.1. Results

**Figure 1:**
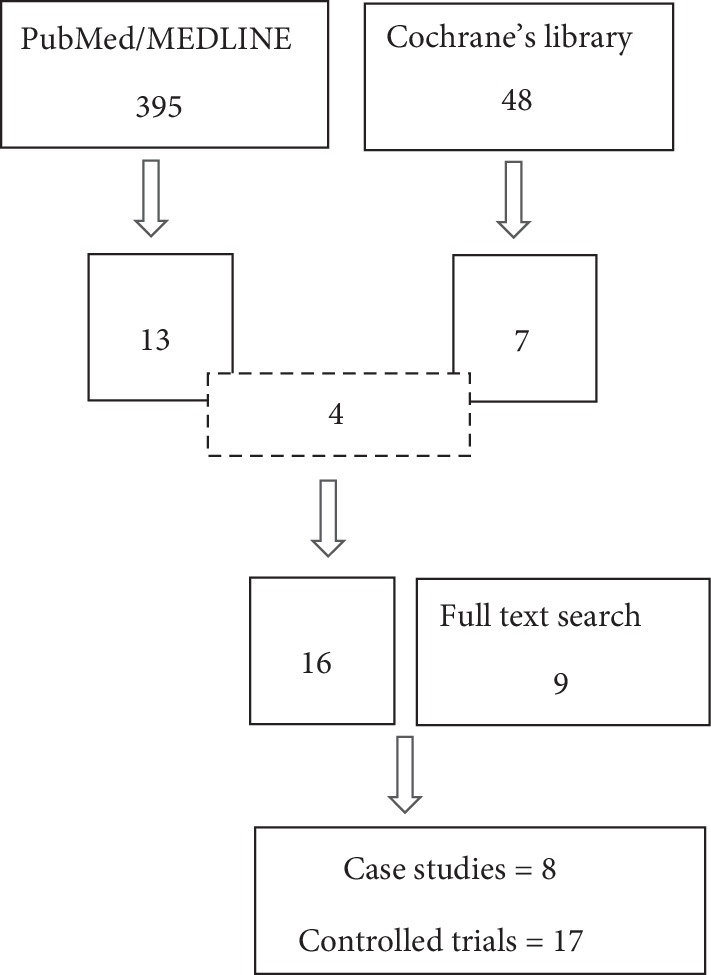
Literature search result.

**Figure 2:**
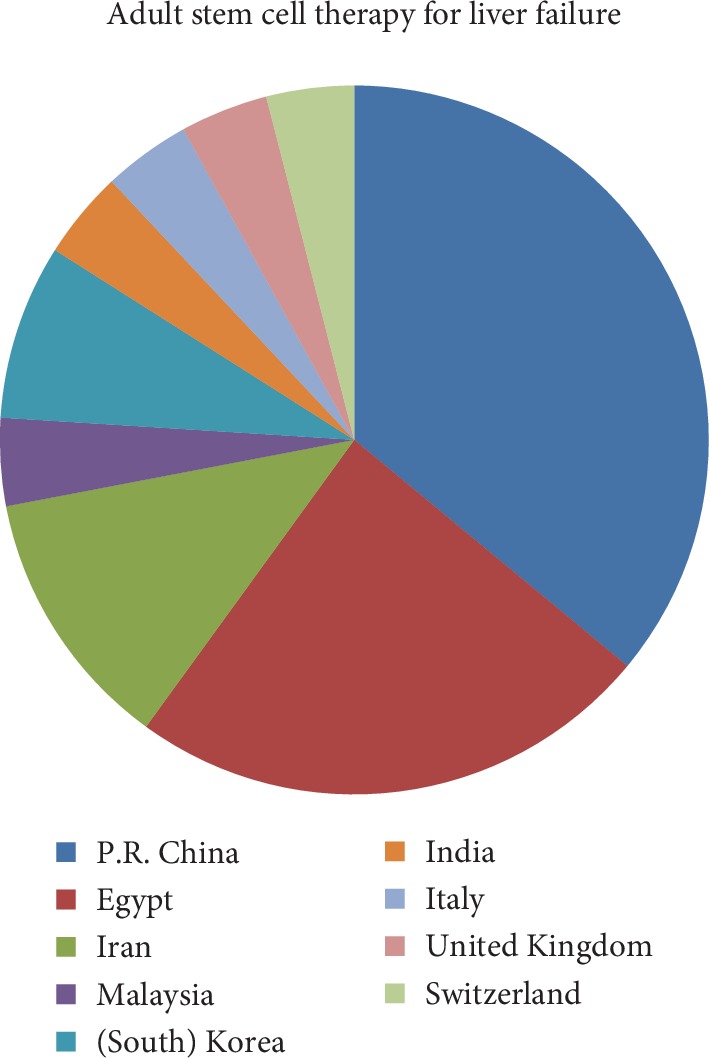
Country distribution of adult stem cell therapy for liver failure.

From PubMed/MEDLINE, we found 395 articles and from Cochrane's library, 48 articles, out of which there were 13 and seven eligible articles from PubMed/MEDLINE and Cochrane's library, respectively, where four articles were found in both (overlapping). In addition, there were nine articles that were found during full-text search. So altogether, there were 25 original articles (Figure 1). Of these articles, 8 studies did not have controls and were classified as case studies (Table 1) [5–12], while 17 studies had controls (randomized and non-randomized clinical trials Table 2) [13–29].  Figure 2 shows country distribution of adult stem cell therapy for liver failure.

### 3.2. Discussion

The eight case studies included variable numbers of patients, from one to 17 patients, and mostly (seven case studies) used various autologous stem cells, i.e., bone marrow- (BM-) mesenchymal stem cells (MSCs) (3 studies) and the rest used peripheral blood (PB) CD133, PB-CD34, CD34-depleted BM-mononuclear cells (MNCs), or BM-CD34 [5–11]. Only one study used allogeneic umbilical cord- (UC-) MSCs [12]. Moreover, there were 17 studies that included 25 to 158 cases and compared stem cell treatments to controls, which used various autologous stem cells, i.e., BM-MSCs (six studies) [13–18], BM-MSC-derived hepatic lineage (one study) [19], BM-MNCs/BM CD133 [20], BM CD34 and CD133 [21], BM-MNCs [22], PB-CD34 (two studies) [23, 24], and allogeneic stem cells, i.e., BM-MSCs [25], UC-MSCs (three studies) [26–28], and stem cell containing umbilical cord blood (UCB) [29]. Moreover, inclusion and exclusion criteria, cell dose, route of administration, concomitant therapies, and outcome measures differed between those studies, which caused a systematic review was more appropriate than meta-analysis.

### 3.3. Safety of Adult Stem Cell Therapy in Liver Failure

Case studies without G-CSF administration and/or leucapheresis did not show any serious adverse events that were related to stem cell therapy [5–7, 11, 12] (Table 1), the same applied to controlled studies without G-CSF administration, where cell therapy-related adverse events were recorded and adverse events in treatments were equal or less compared to controls [16–19]. Further, there was no stem cell-related adverse event in the use of allogeneic MSCs [25–28], and stem cell containing UCB [29] (Table 2). However, a controlled study showed that one out of ten cases who received BM-MNC administration developed hepatocellular carcinoma, compared to none in the control, or those received CD133 cells [20].

Most case studies that used granulocyte colony-stimulating factor (G-CSF) administration did not show any serious stem cell-related adverse events [9, 10]; and controlled studies with G-CSF administration in treatment arm did not show any difference in adverse event occurrence between treatments and controls [15, 21–24]. However, several case studies that used PB stem cells [8, 9] following G-CSF administrationand CD133 [8] or CD34 collection [9] by leucapheresis showed that all cases either experienced increased model of end-stage liver disease (MELD) score and creatinine after G-CSF administration [8], or decrease platelet count at day-1 that returned to baseline at day-7 after G-CSF administration and leucapheresis [9]. Further, a controlled study that used BM-MNC following G-CSF administration showed acute variceal bleeding and aspiration pneumonia [22].

However, administration of G-CSF in another case study that used BM-CD34 [10] and administration of G-CSF in controlled studies that used BM-MSCs [15], BM-CD34/CD133 [21], and PB-CD34 [23, 24] did not show any G-CSF-related adverse event.

In a case study to assess the safety of various doses, higher doses of thawed PB-CD133 after cryopreservation (150,000, 400,000, and 10^6^/kg body weight (BW)) showed a more worsening condition (encephalopathy, hepatocellular carcinoma, and death compared to lower doses (50,000/kg BW) [8].

In the case studies, only two studies used IV route only [6, 12], while the other six used invasive application to the hepatic artery (four studies) [7, 8, 10, 11], or to splenic/IV (one study) [5]) or to the hepatic artery/portal vein (one study) [9].In controlled studies, seven studies used IV route [13–15, 25–27, 29], while other studies used invasive application via the hepatic artery (six studies) [16–18, 22, 24, 28], intrasplenic/intrahepatic (one study) [19], intraportal (two studies) [20, 21], and intraportal/intrahepatic [23]. There was no serious adverse event that was related to invasive route of application. Though IV is the easiest and safest way of administration and does not need special expertise in choosing the most effective way of administration, the results need to be considered. However, comparing the results of various routes of applications was inappropriate, as the studies used different types and/or dose of stem cells, except for two case series that compared either intravenous (IV) and splenic vein application [5] or the portal vein and hepatic artery application [9], and a controlled study that compared intrasplenic and intrahepatic route [19]. In a case series and a controlled study, compared applications showed more or less the same improvement [5, 19]. However, in another case study, one out of three cases, who got PB-CD34 administration through a portal vein showed worsening condition of the liver function, which did not occur in administration through the hepatic artery [9].

### 3.4. Efficacy of Adult Stem Cell Therapy in Liver Failure

Efficacy of stem cell therapy in liver failure was assessed in eight case studies without controls (Table 1) and 17 controlled trials (Table 2). However, case studies without controls lack of rigor, as the improvements might be due to placebo effects, or might be the natural course of disease, as some studies gave additional treatment other that stem cell therapy. Therefore, efficacy is more appropriate to be concluded from controlled trials, and case studies/series are more appropriate for safety studies.

#### 3.4.1. Case Studies/Series Using Adult Stem Cells

Table 1 showed that three case studies transplanted BM-MSCs at various passages (P0 to P4), and various numbers of BM-MSCs, i.e., from 10 × 10^6^ (total number) to 2 × 10^6^/kg BW [5–7]. Most BM-MSC studies showed some improvements; one case series with application via splenic vein or IV [5] was followed up until 6 months and showed improvements in almost all parameters, including decrease in MELD score in part of the cases. Another case series [6] with IV application was followed up until 12 months and showed improvements in albumin level, including MELD score that were preserved until 12 months in half of the cases, while a case report [7] with application to the hepatic artery showed improvement at week-8, but then at week-26 onward improvement decreased and died 12 months after stem cell transplantation.

A case study that used PB CD133 stem cells showed that some cases of all doses experienced a decrease in MELD score and total bilirubin only between wk-1 to wk-6 [8], while another case study that used PB CD 34 showed that some cases of all doses experienced serum bilirubin reduction and albumin level increase [9].

A case study that used BM-CD34, where all cases were followed up until 6 months showed some improvement in some though not all parameters, and at month-6 all cases showed reduced Child-Turcotte-Pugh (CTP) score, and 75% of cases showed reduced MELD score [10].

A case study that used CD34-depleted BM-MNC that was followed up until 12 months showed that the best result was improvement in the albumin level of all cases, but only until day-14, which improvement gradually decreased, except in one out of three remaining cases who also showed decreased CTP score at month-12 [11].

A study that used allogeneic UC-MSCs that was followed up until 12 months showed that improvement was only on gamma glutamyl transferase (GGT) and alkaline phosphatase (ALP) level that occurred in all cases and reduced ascites in all four ascites cases until 12 months [12].

#### 3.4.2. Clinical Trials with Controls Using Adult Stem Cells

Table 2 showed that from 17 controlled trials, a randomized controlled trial (RCT) that gave 120–295 × 10^6^ BM-MSCs via IV route showed no beneficial effect of stem cell therapy compared to control [13], but two other (phase-2 controlled trial and RCT) that gave the same cells and via the same route showed improvement in some of the parameters including reduced MELD Score [14], or CTP score [15]. Both studies that showed improvement used lower number of cells, i.e., 10^6^ cells/kg BW. The three studies using BM-MSCs via IV route differed in inclusion criteria of the cases, where the first study was on post hepatitis B liver failure [13], the second was on hepatatitis C virus- (HCV-) genotype 4 advanced cirrhosis with CTP score C and MELD score > 12 and was given PEG-IFN and Ribavarin [14], and the third was on end-stage liver disease, WHO performance score < 2 [15].Severity of disease and cell number might play a role in the result, where stem cell therapy might benefit for less severe disease, and too many cells might be deleterious. Moreover, concomitant therapy that aimed at the cause, such as the use of antiviral, might be beneficial.

Further, a matched controlled trial and two RCTs gave a dose of 10 × 10^6^, 50 × 10^6^, and around 1 × 10^6^ BM-MSCs, respectively, via the hepatic artery [16–18], and two out of the three trials on post hepatitis B liver failure that was concomitantly given standard supportive therapy and hepatitis B liver cirrhosis that was given entecavir showed improvement in most of the parameters including MELD score decrease [16, 18], while the other one on alcoholic cirrhosis with CTP score B or C and alcohol abstinence only showed improvement in CTP score and fibrosis [17].These three trials showed that concomitant therapy to stem cell therapy might be beneficial to enhance the effect of cell therapy.

An RCT on end-stage liver disease due to hepatitis C virus (HCV) with CTP grade C, serum albumin < 2.5 mg/dl, prothrombin concentration (PC) < 60%, and MELD score < 25 used BM-MSC-derived hepatic lineage (20 × 10^6^ hepatic lineage cells in 200 × 10^6^ BM-MSCs) that was given intrahepatic or intrasplenic showed no difference between the two routes, and showed partial improvement including CTP and MELD score compared to controls [19].

Two RCTs that used BM-MNCs with a total dose of 1000 × 10^6^ and a dose of 50 × 10^6^/kg BW, which was given intraportal and the hepatic artery, respectively, showed no benefit compared to control [20, 22], but when BM-MNCs were enriched for CD133 with a dose of around 5 × 10^6^ [20] or CD34/CD133 with a dose of 55 × 10^6^ [21], there were partial improvements [20, 21]. It seems that using too many BM-MNCs intraportal or intrahepatic is counterproductive, but when they are enriched to reduce the cell number, they are more beneficial. Two non-RCTs that used CD34-enriched PB-MNCs with a dose of 1000 × 10^6^ and 20-40 × 10^6^ that were given intraportal or the hepatic artery showed partial improvements compared to controls [23, 24].

Four studies that used allogeneic MSCs [25–28] and an RCT that used umbilical cord blood (UCB) [29] showed various grades of improvements. An RCT on acute on chronic liver failure (ACLF) with MELD score 17-30 used cryopreserved allogeneic BM-MSCs with a dose of 1-10 × 10^5^/kg BW via IV route and concomitant standard therapy showed improvements in some parameters including MELD score [25]. Three controlled trials used fresh [26, 28] or cryopreserved [27] UC-MSCs with a dose of 5 × 10^5^/kg BW via IV route [26], a dose of 450 × 10^6^ via IV route [27], or a dose of 200 × 10^6^ via the hepatic artery [28], all with concomitant standard therapy, where two controlled trials showed improvements in most parameters [27, 28], while another one showed improvements in only some of the parameters including MELD score [26]. Allogeneic use of stem cells might need to be cryopreserved to match the availability of cells and patients, and the studies above showed that cryopreserved cells might be equal to fresh cells [27, 28], and both IV or invasive application via the hepatic artery might be equal [27, 28].

One RCT used stem cell containing UCB compared with blood transfusion once to three times a week, with a total transfusions of two to five times, and showed partial improvements compared to control [29].

### 3.5. Animal Studies on Stem Cell Therapy for Liver Failure/Cirrhosis

Various animal study showed beneficial effect of adult stem cells to treat liver damage and failure [30–33].

A study used bone marrow-derived mesenchymal stem cells (BM-MSCs) infusion to treat ConA-induced liver damage in mice showed that the BM-MSCs were found in the liver and liver damage was reduced. Moreover, the transplanted BM-MSCs caused improvement in immune function by suppression of intrahepatic natural killer T cells, which previously induced liver damage upon ConA treatment [30]. Another study used intraparenchymal liver injection of human or rat adipose-derived mesenchymal stem cells (AD-MSCs) to treat rat models of acute-on-chronic liver failure and showed improvements in liver failure symptoms including ascites, hepato- and splenomegaly, and serum liver biochemical parameters [31]. A recent study used BM-MSCs and colony-stimulating factor-1-induced bone marrow-derived macrophages (id-BMMs), either alone or in combination, to treat carbon tetrachloride- (CCl4-) induced cirrhosis in mice. All kinds of treatment showed beneficial effects in terms of liver fibrosis reduction, which was due to matrix metalloproteinase (MMP-9, MMP-13) secretion, decrease in liver enzyme levels in blood, and increase in hepatocyte proliferation, but the most effective treatment was obtained by combination therapy [32]. Moreover, a study compared intravenous and intrasplenic administration of BM-MSCs to CCL4 induced liver fibrosis in rats and found that both routes showed similar improvements in liver functions, but intravenous route showed better reduction in IL-1*β*, IL-6, and INF-*γ* [33].

### 3.6. Pluripotent Stem Cell-Derived Cell Therapy for Liver Failure/Cirrhosis

To regenerate damaged liver tissue, hepatocytes are an option for cell therapy. As hepatocytes cannot be expanded in vitro, production of hepatocytes from infinitely self-renewable pluripotent stem cells (PSCs), namely embryonic stem cells (ESCs) or induced pluripotent stem cells (iPSCs) that were initially developed by Takahashi and Yamanaka [34], is an important solution. Many studies developed protocols to induce PSCs into hepatocytes, though most of the hepatocytes were more similar to immature hepatocytes, and therefore were called hepatocyte-like cells (HLCs) [35] Some of the studies used combination of growth factors that were given in a sequential manner [36, 37], or combination of transduction factors (FOXA2 and HNF1alpha) [38]. In addition, a study used extracellular matrix laminin to induce ESCs into HLCs [39], and another study used a chemically defined culture medium to induce ESCs into HLCs [40].

Recently, a study succeeded to develop hepatocytes from human pluripotent stem cells in a current good manufacturing practice (cGMP) setting that are compliant for cell therapy. The study used two human iPSC and one ESC cGMP lines to generate functional hepatocytes using a highly reproducible protocol. The protocol used chemically defined media and consisted of four steps to produce immature progenitor of hepatocytes in 21 days, followed by seeding the immature progenitor on a scaffold made from 3D poly(ethylene glycol)-diacrylate hydrogel lattice to induce the maturation into hepatocytes. These hepatocytes were tested on immune-competent mice and were showed to be viable and functional [41].

### 3.7. Mechanism of Action of Liver Failure Healing in Adult Stem Cell Therapy

From the various kinds of stem cells, most studies using MSCs either from the bone marrow [14–16, 18, 25] or umbilical cord [26–28] showed the most promising results, especially when combined with concomitant standard/supporting therapy [14, 16, 18, 25–28].

MSCs are stromal cells that can be differentiated into hepatic lineage [19], though most studies showed that in stem cell therapy, most stem cells did not differentiate into the needed cells to replace damaged cells except in several conditions such as bone repair [42]; instead stem cells mostly work through paracrine effect [42]. MSC paracrine effect was shown to target hepatic stellate cells to reduce fibrosis [43], and this effect was also shown in a randomized controlled trial that found 25% and 37% reduction of fibrosis in treatment groups that received autologous BM-MSCs in a dose of 5 × 10^7^ cells via the hepatic artery as once and twice administration, respectively [17].

This review showed that IV route was equal to invasive application via the hepatic artery [19, 27, 28] or the splenic artery [5] and was safer compared to portal vein delivery [9] that deliver the stem cells directly to the liver. These findings might be due to the fact that when MSCs are delivered via IV route, after 30 minutes they are trapped in the liver, spleen, and lungs, and after 90 minutes they are in the liver, spleen, and bone marrow [44]. When MSCs home to liver, they can send mitochondria-containing tunneling nanotubes to their surrounding damaged hepatocytes to repair the damage [42]. For MSCs that are trapped in other area/organs other than liver, they can communicate with the damaged hepatocytes in the liver from afar by means of extracellular vesicles (microvesicles and exosomes) and send beneficial factors such as cytokines and growth factors including hepatocyte growth factors (HGF) to heal the damaged hepatocytes [42, 45] or to act on resident stem cells to differentiate and to replace the damaged hepatocytes.

Both autologous [14–16, 18] and allogeneic [25–28] MSCs were beneficial and did not show any cell-related adverse event. MSCs lack human leucocyte antigen (HLA)-DR and T cell costimulatory molecules [46, 47]; therefore, allogeneic MSCs are regarded relatively safe for large number systemic infusion even they are not HLA matched.

Overall, none of the studies showed a complete and sustainable performance in the outcome measures. Moreover, various types of cells, various doses, and various routes were used, which need more studies on the quality of the cells, as well as the safety and efficacy of the therapy in well-designed clinical trials that is compliant with government regulation for cell therapy in the countries where the trials are conducted.

## 4. Conclusion

Among the various adult stem cells that were used in human studies, MSCs from the bone marrow or umbilical cord performed better compared to other types of adult stem cells, though no study showed a complete and sustainable performance in the outcome measures. IV route was equal to invasive route. Fresh or cryopreserved, and autologous or allogeneic MSCs were equally beneficial; and giving too many cells via intraportal or the hepatic artery might be counterproductive.

## Figures and Tables

**Table 1:** Case studies on stem cells for liver failure [5–12].

Ref no	Stem cell type passage dose	Route	Severity/other concomitant therapy	Study, P-no	Monitoring-outcome measures	Outcome
5	T1: auto BM-MSCs–P0-10 × 10^6^–in 5 mL saline T2: auto BM-MSCs–P0-10 × 10^6^–in 5 mL saline	T1: splenic vein-CT guidance T2: IV	Chronic hepatic failure, liver cirrhosis-CTP grade C, MELD score > 12/(-)	Phase I trial, T1 = 6 (4 M) T2 = 6 (5 M)	BL, mo-1-2-3-4-5-6: encephalopathy, jaundice, hematemesis/melena, LL edema, ascites, ALB, TBil, DBil, SGOT, SGPT, GGT, PC, Cr, MELD score	Mo-ND: no-encephalopathy ↓T1 (2/3), T2 (1/2), jaundice↓ T1 (2/5), T2 (1/4), LL edema↓ T1 (3/5) T2 (2/4), ascites↓T1 (2/3), T2 (2/3) T1 and T2: TBil↓, dBil↓, Cr↓, MELD score↓ T2: SGOT↓

6	T = vancomycin IV, h-1-auto BM-MSC P2-P4, mean: 31.73 (10.2‐60) × 10^6^, viability 95%, diluted in 20 mL normal saline	IV–heparinized syringe–30 minutes	Chronic liver failure, MELD score ≥ 16/diuretic (4/4), AIH medication (1/4)	*T* = 4 (1 M)	BL, d-1-4-7, wk-2-3-4, mo-2-3-6-9-12: AE, physical exam, CBC, PT, INR, s-urea, Cr, ALB, ALT, AST, ALP, TBil, dBil, AFP BL, mo-6: CT scan-liver vol, QoL, MELD score	AE (-) mo-6-12: edema↓ (4/4), diuretic dose↓ (1/4), mo-6: ALB↑ (3/4) mo12: ALB↑ (2/4) mo-6: liver vol↑ (3/4) QoL↑(4/4) mo-6-12: MELD score↓ (2/4)

7	T =auto BM-MSCs, P4, 2x10^6^/kgBW, in saline, Vvol: ND, 2x, interval 40 days	Hepatic A	Decompensated alcoholic liver cirrhosis, CTP grade C	*T* = 1 (M)	BL, wk-6, wk 8, wk 26, wk 52: TBil, s-Alb, Cr, INR, CTP grade, diuretic use, ascites	Wk 8: TBil↓, s-Alb↓, Cr↑, INR ↓, CTP score↓, diuretic use↓, ascites↓ Wk 26: TBil↑↓, s-Alb↓, Cr↓, INR ↑, CTP score↑, died 12 mo after transplant

8	Cryopreserved– thawed (at least 4 wk) auto PB CD 133 (at least 4wk after leucapheresis) T1 = 50,000, T2 = 150,000, T3 = 400,000, T4 = 10^6^/kgBW In 30 ml saline-10% human albumin-10% acid citrate dextrose (anticoagulant)	Via hepatic artery–in 3 minutes	End-stage liver disease-MELD score = 17-25, CTP B or C/ Rec hu G-CSF (Lenograstim Italfarmaco)-7.5 *μ*g/kgBW-bid-SC-5-7 days–leucapheresis from d-4 if auto PB CD 133 ≥ 8/*μ*l After auto PB CD133: GCSF 5 *μ*g/kg BW–3 days	Phase-1 trial, Total = 17 T1 = 3 (P1-2-3) T2 = 3 (P6-7-9) T3 = 3 (P10-11-12) T4 = 3 (P13-15-17)	During GCSF treatment: BL-d-3-4-5-6-7: PB CD 133 monitoring–no of CD 133 collected, MELD score, AE, Tbil, Cr, INR ReI, d-1-2-3-4-5-6-7-wk-2-3-4, mo2-3-6-9-12 mo: physical examination, Tbil, Cr, INR, ALB, MELD score	Withdraw: P-16 Not enough CD 133 = 2 (P4-14), after GCSF- MELD score↑ (12/12), sig↑ = 2 (CTP-grade C), TBil↑, INR↑, Cr↑ (12/12),hepatorenalsyndr =2 (P-5: died, P8: OLT) ➔enrolled =12 AE: hematoma=2, worsening condition = 3 (i.e., encephalopathy = 2 (d-3-PR-P-10, d-30-DP-P17), HCC nodule = 1-P-15), OLT = 5 (within 12 mo, P-1-6-12-13-17), LTF➔ died = 2 (P-7-10)- (mo 5, mo 6), d-1-3: med WBC↑, wk-1-mo-1-2-3-6: med MELD score↓, med TBil↓, mo-1-2-3-6: med INR↓, mo-1-2-6: med Cr↑, mo-3: med Alb ↑(10/12)

9	T (after leucapheresis) = auto PB CD34+ 10^6^ (P1-P3-P5), 2 × 10^8^ (P2-P4)–in 20 mL normal saline	To portal vein (P1-P2-P3) or hepatic artery (P4-P5)	Chronic liver failure, WHO performance status < 2/ G-CSF (G-CSF, Chugai Pharmaceutical)-526 *μ*g SC-daily–5 days, leucapheresis	T = 5 (4 M)	d-1: AE BL-d-7-15-45-60, every 2 mo until 6 mo (P3), 12mo (P1-P2-P4), 18 mo (P5): AE, physical exam, CBC, liver function (ALB, Bil), urea, coagulation profile d-60: AFP, abdomen CT scan	d-1: PC↓ (all), but returned to baseline on d-7, nausea (5/5), pain at site (4/5), fever (1/5), vomiting (1/5), rash (1/5). P-1: mo-1-2 s-Bil↓ = N, mo-6-to 12↑but < BL P-2: mo-1 to12 - Bil↓ = N P-3: Bil↑, ALB↓ P-4:-mo-6-AE: severe UTI- AB, s-Bil↓ (50% BL) mo12 –s-Bil = BL) P5: mo-1 to 12 s-Bil↓, mo 18 ↑ = 50% BL ALB↑(slightly)–through FUP–all except P-3

10	T = auto BM CD34+ 10x10^6^, purity = 95%, viability = 92.5% in 10 mL normal saline	To the hepatic arter–1 mL/minutes	Chronic liver failure/G-CSF (ND) 300 *μ*g/mL SC daily–5 days	*T* = 4	d 1-2-3-4-5: AE (fever, chills, hives, angina pectoris) BL-d7-15, mo-1-2-3-4-5-6: ALB, ALT, AST, Bil, s-HA, Cr, CTP, MELD score	AE (-) mo 2-3-4-5-6: P1-2: ALB↑, ALT↓, AST↓ mo 1-2-3-4-5-6: P3-4: Bil↓ mo-6–P1-2-3-4: HA↓, CTP score↓mo 6 – P1-2-3: MELD score↓

11	T = auto CD34 depleted BM-MNC by CliniMACS-plus-fresh–cell number: ND, vehicle: ND, V vol: 310-410 (med = 355) mL	Via hepatic artery–1200 mL/hour	Liver failure waiting for OLT/standard therapy	T = 5 (2 M)	BL -d-0-7-14, mo-1-2-3-4-12: **AE**, s-ALB, TBil, DBil, PT, AST, ALT, s-AlP, s-HA, **CTP score**, LE, subj H, **QoL** Mo-2-4-6-8-10-12: CT scan **(**ascites, **liver volume**, HCC)	Serious AE (-), **P-1–OLT at mo-4, P-3–OLT at wk-3** d-7-14: s-ALB> BL (5/5), INR↓: d-14 (3/5), mo-1-2 (2/4), mo-3-4 (1/4), EL↓: wk-4 (3/4), wk-8 (4/4), wk-16 (1/2), mo-1-2-3-4-5-6: ascites ↓ (2/4) mo-12**: CTP score** ↓ (1/3), **QoL**↑ (2/3), ↓(1/3), **liver vol**↓(3/4), ↑(1/4) subj H: D1-Mo1-↑ (4/4), mo-2 ↑ (3/4), mo-3↑(2/4), mo-4↑(1/4)

12	T = UC-MSCs-P4, 0.5 × 10^6^/kgBW in saline–Vvol: ND	IV–3x interval 4 wk	Primary biliary cirrhosis–incomplete response to UDCA/standard medication (UDCA)	*T* = 7 (1 M)	AE: short term, long term. BL, wk-24-48: Symptoms (pruritus, fatigue, fever, peripheral edema, rash, nausea, vomiting), physical exam, liver function (ALT, AST, GGT, ALP, ALB, TBil, PTA), INR, QoL, USG-hypogastric ascites, Mayo RS, MELD score	AE short term: self-limiting fever (1/7) Wk 48: pruritus↓(5/5), fatigue↓(7/7), ALP↓(7/7), GGT↓(7/7), BL vs wk 48: ALP↓ (474.29 ± 223.26 vs 369.86 ± 168.35 IU/L, *P* = 0.044), GGT↓ (194 ± 140.65 vs 132.71 ± 129.4 IU/L, *P* = 0.049) wk-24-wk 48: hypogastric ascites↓ (4/4) wk 48: Mayo RS↑ (4/7), MELD score ↑(2/7), ↓(1/7)

Auto = autologous, BM = bone marrow, MSCs = mesenchymal stem cells, P0 = passage-0. CT=, IV = intranevous. Rec = recombinant, hu = human, GCSF = granulocyte colony stimulating factor, BW = body weight, bid = twice per day, SC = subcutaneous, d- = day-, PB = peripheral blood, wk = week, MNC = mononuclear cells, V vol = vehicle volume, med = median, h- = hour-, P2 = passage 2, P4 = passage 4. CTP = Child Turcotte Pugh, MELD = model of end-stage liver disease, OLT = orthotopic liver transplantation, AIH = auto immune hepatitis, UDCA = ursodeoxycholic acid. M = male, BL = base line, mo = months, LL = lower limb, ALB = albumin, Tbil = total bilirubin, Dbil = direct bilirubin, SGOT = serum glutamic oxaloacetic transaminase = AST, SGPT = serum glutamic pyruvic transaminase = ALT, GGT = gamma glutamyl transferase, PC = prothrombin concentration, AE = adverse reaction, Cr = creatinin, INR = international normalized ratio, ReI = reinfusion, QoL = quality of life, subj H = subjective healthiness, ALT = alanine aminotransferase, AST = aspartate aminotransferase, s- = serum, AlP = alkaline phosphatase, HA = hyaluronic acid, LE = liver elastography (measuring liver stiffness), CBC = complete blood count, AFP = alpha fetoprotein, PTA = prothrombin time activity, RS = risk score, USG = ultrasonography. ND = no data, no- = number of cases with – (remaining cases/base line number), Sig = significant, PR = promptly resolved, DP = disease progression, LTF = lost to follow up, P- = patient-, N = normal, FUP = follow up period, vol = volume, med = median, WBC = white blood count, HCC = hepatocellular carcinoma.

**Table 2:** Controlled studies on stem cells for liver failure [13–29].

Ref no	Stem cell type passage dose,	Route	Severity/other concomitant therapy	Study, P-no	Monitoring-outcome measures	Outcome
13	T: auto BM-MSC, P3-P4 median = 195 × 10^6^ (120-295 × 10^6^)-viability > 95%, vehicle: saline, vol: 100 mL C: saline 100 mL-	T: IV in 30 minutes C: IV- in 30 minutes	Decompensated cirrhosis/(-)	RCT *T* = 15 *C* = 12	BL-24 h, 1-3-6-12 mo: AE/SE: NE, MELD score, CTP score, liver function (ALB, ALT, AST, TBil, INR), PT, s-Cr, liver volume, survival	AE: NE 12 mo: T: 3/15 died C: 0/12 died T vs C: MELD score, CP score, ALB, ALT, AST, INR, liver volume –NS T and C: no HCC

14	T1 = auto BM-MSC-P5 T2 = auto BM-MSC-diff hepatocytes (40% HLC-60% MSC) T1 = T2 = 1 M/kgBW-1 M/mL–saline- C = (-)	T1 = T2: IV in 15 minutes-5 drops/min	HCV-genotype 4, advanced cirrhosis-CTP-C, MELD > 12/PEG-IFN, Ribavarin	Phase II trial T1 = 9 T2 = 6 *C* = 10	AE, SE: NE BL, mo-3, mo-6:Enc, Jau, Hem/Mel, LL-Ed, Asc, Eryt, Tr, itch, PC, Hb, WBC, PlC, Cr, Alb, SGOT, SGPT, AlP, TBil,DBil, IBil, GGT, AFP, MELD score	AE: NE Enc, Jau, Hem/Mel, LL-Ed, Asc: BL: T1 = T2 = C Mo-3: T1 = T2 < C (*p* < 0.001, <0.001, 0.005, <0.001, 0.061) Mo-6: T1 = T2 < C (*p* = 0.002, <0.001, 0.005, <0.001, 0.008 Alb, PC, Hb: BL: T1 = T2 = C Mo-3: T1 = T2 > C (P = 0.002, 0.048, 0.002) Mo-6: T1 = T2 > C (*P* = 0.002, 0.023, 0.014) TBil, IBil, MELD score: BL: T1 = T2 = C mo-3: T1 = T2 < C (*P* = 0.01, 0.003, 0.014) mo-6: T1 = T2 < C (*P* = 0.004, <0.001, 0.003)

15	T: G-CSF (Neupogen, Roche)-300 *μ*g SC –5d+auto BM-MSCs–P0-1 × 10^6^/kg BW–vehicle: ND, volume: ND C: SMT: ND	IV	End-stage liver disease, WHO performance score < 2/(-)	RCT, *T* = 20 *C* = 20	BL-every hour-24 h-1-2-3-4-wk-2-3-4-5-6 mo: SE (fever, immune reaction, USG, Doppler, AFP),ascites, LL edema, s-bil, ALB, PT, PC, INR, ALT, AST, bU, sC, FBG, BGAM, CTP score, pcoll III,	SE: (-) 3-6 mo–T: ALB↑, INR↓, PC↑, ALT↓. AST↓, CTPscore↓, pcoll III↓ 0/20 died C: no improvement, 5/20 died

16	T = auto BM-MSCs – P3, 10^6^ cells/mL-in 10 mL normal saline C = (-)	To hepatic arter–over 20-30 minutes.	Post hepatitis B liver failure/SMT1	Matched *T* = 53 *C* = 105	BL-short term (wk 1-2-3-4): AE, complication, S-RS, HoS, ALT, ALB, Tbil, PT, MELD score Long term (wk4- 48): ALT, ALB, Tbil, PT, MELD score Long term prognosis (wk 4 until-wk 192, every 12 wk): HCC incidence, survival/mortality rates	Adverse event (-) Wk 2-24: ALB↑ T > C, Wk2-12: Tbil ↓ T > C, wk 3-12: PT ↓ T > C, wk 3-36: MELD score ↓ T > C Mean HoS: T ≈ C wk 192: HCC, mortality T ≈ C

17	T = auto BM-MSC, P4-P5, 5 × 10^7^, in 10 mL plasma solution A T1 = one time (1mo after aspiration) T2 = 2 times (1mo, 2 mo after aspiration), 2mo cryo at P1 C = SMT: ND	To hepatic artery	Alcoholic cirrhosis, CTP score B-C/alcohol abstinence 6 mo before till mo12	RCT T1 = 18 − 1 = 17 T2 = 19 − 2 = 17 *C* = 18 − 2 = 16	BL, every wk➔wk 52: AE BL-mo3-4-5-6-7-8-9-10-11-12: liver function: AST, ALT, Alb, Bil, ALP, GGT, BUN, Cr, INR, AFP, CEA, PT, BG, TG, TChol, CTP score, MELD score BL- mo6: fibrosis	AE: T2: fever 1/19 Other AE: T1 = T2 = C 6mo-fibrosis reduction: T1 = 25%, T2 = 37%, C = none CTP score: baseline➔mo12 T1: 7.6 ± 1.0➔ 6.3 ± 1.3 (SD) T2: 7.8 ± 1.2➔ 6.8 ± 1.6 (SD) C: 8.1 ± 1.3➔ 7.4 ± 1.5 (NSD)

18	T: auto BM-MSC-P3 from 130-150 mL BM, dose: 0.75 ± 0.50 × 10^6^, in 20 mL normal saline–C: (-)	Infused into liver 1 mL/min Via hepatic artery (Seldinger technique)	Hepatitis B liver cirrhosis/antiviral (entecavir 0.5 mg/day)	RCT *T* = 27 − 7 = 20 *C* = 29 − 10 = 19	BL, wk1-2-4-8-12-24: AE, ALT, TBil, Alb, PT, INR, MELD score, HBV DNA, Cr, serum cytokine: TNF-*α*, TGF-*β*, IL-6, IL-1, PB-MNC: Th17, Treg cells	AE: T: fever (< 38.5°C) 4/20 Wk1-2-4: TGF-*β*: T > C Over time: TNF-*α*, IL-6, IL-1: T < C Wk1-2-4-8-12-24: ALT, TBil: T < C wk 2-4-8-12-24: Alb T > C wk 4-8-12-24: MELD score: T < C wk 2-4-12: Treg/T17: T > C

19	T1 = T2: Vit K 3d before transplant, auto BM-MSC➔ hepatic lineage,2 × 10^7^ (in 2 × 10^8^ MSC), in saline, vol = 5 mL, C: SMT-2	T1 = intrasplenic T2 = intrahepatic	End-stage liver disease due to HCV, CTP grade C, s − ALB < 2.5 mg/dl, PC < 60%, MELD score < 25/(-)	RCT *T* = 20 (16 M) ➔T1 = 10, T2 = 10 *C* = 20 (17 M)	BL, wk 2, mo-1-2-4-6: SE, hematology, s-Alb, s-Bil, liver enz, INR, LL edema, Abd USG (ascites), CTP score, MELD score, fatigue scale, performance status	SE: 24 h-fever –antipyretic (T1 = 7, T2 = 3), transient shivering T2 = 3 All parameters: T1 vs T2– NSD, hematology, sBil, liver enz, INR: T vs C- NSD, ascites: T↓ vs C↑-SD wk2-mo 1-2-4, LL edema T↓ vs C-SD wk2-mo1-2-4-6, s-Alb: T↑ vs C –SD wk 2-mo-1-2-4-6, MELD, CTP score, fatigue scale: T↓ vs C↑-SD-wk2-mo 1-2-4-6, performance scale T↑ vs C = -SD wk 2-mo1-2-4-6.

20	T1 = auto BM-MNC(viability > 95%), *I* = 7.62 ± 5.53 × 10^8^, II = 9.17 ± 5.24 × 10^8^–in 20 mL normal saline+2.5% HSA T2 = CD133+ cells (viability > 95%, purity > 85%), I = 4.74 ± 2.61 × 10^6^, II = 9.64 ± 1.75 × 10^6^-in 15-20 mL normal saline+2% HSA C = auto cell-free serum 2x: BL, Mo-3	T1: intraportal T2: intraportal C: intraportal	Decompensated cirrhosis waiting for LT, CTP class B or C/(-)	RCT T1 = 10➔ 8 T2 = 8 ➔ 4 C = 9➔ 6	BL, mo-3, mo-6: AE, INR, Bil, AST, ALT, MELD score mo-12: mortality	AE: (-) T1: HCC = 1, died = 1 (MELD score = 22) T2: LT = 2, LTF = 2 C: LT = 1, LTF = 1, died = 1 (MELD score = 20) Other AE: (-) mo-3: MLD score- T2↓vs BL (NS, *P* = 0.07), INR T2↓vs C↑ (*P* = 0.03), Bil-C↓vs BL (*P* = 0.03) mo-6: Bil-C↓vs BL (P = 0.03), AST – T2↓vs BL(NS, p = 0.06), ALT-T2↓vs BL (p = 0.02)

21	T: G-CSF (Neupogen, Roche)-300 *μ*g-daily-SC-5d, auto BM-CD34, CD133-50 M-in100 mL saline-infusion C: daily SC-distilled water-5d, saline infusion + SMT-ND	Intraportal-ultrasound guidance	End-stage liver disease, WHO performance score < 2/(-)	RCT *T* = 90 *C* = 50	BL- every hour-24 h-wk-1-2-3-4-mo2-3-4-5-6: AE, sBil,Alb, PT, PC, INR, ALT, AST, bU, s-Cr,FBG, BGAM, CBC, CTP score, performance score, Asc,Enc, hem, HRS, survival	AE: T: mild pain, discomfort-infusion site, fever < 24 h = 15/90, transient bone pain = 23/90. 6 mo: ALT↓–*T* = 30/90, *C* = 0/50, AST↓–*T* = 39/90, *C* = 0/50, Alb↑-*T* = 40/90, *C* = 0/50 PC↑-*T* = 48/90, C = 0/50, Bil↓-*T* = 39/90, *C* = 0/50 Asc: disappear *T* = 21/90, *C* = 0/50, reduced–*T* = 51/90, *C* = 0/50 enc(-): *T* = 91.3%, *C* = 19.2% Hem-(-): *T* = 87%, C = 23% CTP imp: T = 48/90, C = 0/50 Died: T = 9/90 (hem = 7, HRS = 2), C = 26/50 (hem = 15, HRS = 5, Enc = 6)

22	T = G-CSF (Lenograstim, Sanofi Aventis)10 *μ*g/kg BW–SC-5 d, BM-MNC (from 103 ± 18 mL BM) = 47 ± 15 × 10^6^/kg, CD34 = 24 ± 11 × 10^4^/kg, MSC = 34 ± 59 × 10^4^,in 80 mL NaCl-75% hu albumin-5% CSL-heparin 10 U/mL-C = (-)	To hepatic artery-in5 minutes	Decompensated alcoholic liver disease, MELD < 26/SMT3	RCT *T* = 28 *C* = 30	BL, wk 4, wk 8, wk 12: AE; plasma TNF*α*, TNF*α*R1, IL6, AFP, HGF, TGF*β*; blood ethanol level. BL, wk4: biopsy-Ki67/CK7 HPC wk 12: MELD score	AE: *T* = 17/28, *C* = 24/30 During G-CSF: T = 2 (acute variceal bleeding, and aspiration pneumonia) Died: *T* = 2 (acute variceal bleeding, liver failure), *C* = 4 (intracranial haemorrhage, sepsis (2), multiple organ failure) Wk12: MELD score ↓ ≥3–*T* = 64%, *C* = 53% (*P* value: 0.43, OR = 1.6, CI = 0.49–5.4) Wk12: cytokine: T vs C: NSD

23	T = G-CSF 300 g (Neupogen, Roche) SC, daily–5d, LP, expanded PB-CD34 MNC–1 × 10^9^ in physiologic saline (volume: ND) C = SMT-4	To the portal vein (if hepatopedal flow) or hepatic artery (if hepatofugal flow)	Post hepatitis C advanced cirrhosis, WHO performance score < 2 –/(-)	Allocation: atient preference, *T* = 50, *C* = 50	BL-mo 1-3-6-12: AE, SE: NE. Complication, HR- QOL (questionnaire), ascites, survival time, 1y-survival, CBC, AST, ALT, ALP, s-Bil, PC, INR, s-Cr, abdominal USG	AE: NE Complication: C > T Mo 1-3-6-12: HR-QOL T > C, ascites grade ↓ (T > C), mean survival time: *T* = 359.3 days, *C* = 277.56 days, 1y-survival: *T* = 94%, *C* = 62%

24	T = G-CSF (ND) -5-10 *μ*g/kg BW-QD-SC-4d + auto PB CD34SC- 2-4 × 10^7^, vehicle: ND, volume: ND C = (-)	To hepatic artery	Decompensated liver cirrhosis, CTP = grade B and C (*T* = 8.43 ± 1.04, *C* = 8.07 ± 1.36)-T vs C -NS /SMT-5+ HGF IV drip (2 wk)	Controlled study, *T* = 23 (14 M) *C* = 28 (17 M)	BL-4-12-24-36-48 wk: AE, symptoms: fatique, anorexia, abdominal distension, ascites; lab: ALT, AST, TBIL, ALB, PTA; CTP score, liver tumor (USG) BL-12-24-36-48 wk: ICG-R15	SE: T: mild fever (2/23) -2d - resolved T: symptom improvement T: ICG-R15↓ T-C 48 wk: CTP↓ (decrease T > C) 48 wk-AscR: *T* = 9/23, *C* = 8/23 48 wk-AscD: *T* = 7/28, *C* = 8/28 T-C: ALT, AST, TBIL - no change T-C: ALB↑ (T > C) T-C: PTA↑ (T > C)

25	T: Allo BM-MSCs cryopreserved at P5-P6, thawed-washed, 1-10 × 10^5^/kgBW––in 50 mL saline +10 mL saline-1x/wk –4wk C: (-)	IV–infusion –in 30 minutes	ACLF, MELD score 17 − 30/SMT-6	RCT, *T* = 56 *C* = 54	BL, immediate after infusion, 1-2-3-4-8-12-24 wk: AE (fever, rash, diarrhea), WBC, Hb, PlC, creatinine, HCC, tumor ALT, ALB, TBil, INR, MELD score, survival time, liver failure-related complication	Adverse event: fever- *T* = 19.2%, C- 2.4% Died: T: 15/56, C: 24/54, FDI: *T* = 19.2%, *C* = 2.4%. Wk-1: ALT, ALB improvement T > C wk-1, wk-2: MELD score ↓ T> > C 1^st^ 4 wk, entire 24 wk: TBil, MELD score↓ T > C

26	T: fresh hu UC MSCs-P4 500.000/kg BW-in saline-volume: ND 3x–4wk interval - C: saline-equal volume to T	T: IV C: IV	ACLF, MELD score = 24 ± 4 (T), 26 ± 4(C)/SMT-7	OLPC, PhI/II, *T* = 24 *C* = 19	BL-1-2-4-8-12-24-36-48 wk: AE, symptoms: fever, edema, rash, nausea, vomiting; lab: ALT, TBIL, ALB, CHE,PTA,PC; MELD score, 72 wk: SA	SE: T: mild fever (2/24)–self-limiting-12 h T: ALB↑, PC↑ T-C: CHE↑, PTA↑ (T > C) T-C: ALT↓ (T = C), TBIL↓ (T < C) T-C: MELD score↓ (T < C) T: survival rate↑

27	T: hUCMSCs–cryopreserved-P3–(4.0–4.5) × 10^8^–in 100 mL normal saline- on day-3 and day-4 C: (-)	IV-slowly–in less than 1 hour	Decompensated liver cirrhosis due to hepatitis B/SMT-8	RCT *T* = 50 matched *C* = 53	BL- 2-4-8-12-24-36 wk: AE, IL 6, TNF-*α*,IL10, TGF-*β*,T4, Treg, T8, B cells, ALT, AST, ALB, TBil,PT,MELD, CTP score, LF, MR	Adverse event (-) 2-4 wk: IL-6, TNF-*α* (T↓ > C), IL-10 (T↑ > C) 2 wk: TGF-*β* (T↑ > C) 2-4 wk: T4, Treg (T > C), T8, B cell (T < C) 8-12 wk: AST-(T↓↓ > C) 4-8-12-wk: ALB (T↑ > C), TBil(T↓ > C) 2-4-8-12 wk: PT (T↓ > C) 4-8-12-24-36 wk: MELD, CTP score (T↓ > C) LF: *T* = 1, *C* = 5 MR: T < C

28	T: after 4-7 session PE–fresh UC-MSCs –P3-P4- 2× 100 × 10^6^ in 2× 60 mL normal saline C: (-)	To hepatic artery–in 15 minutes	HBVrACLF/ entecavir +PE 2-3x/wk + SMT-9	Consecutive, *T* = 11 *C* = 34	BL-daily-2-4-8-12-24-48-60-72-84-96 wk: AE: HCC and mortality (24mo survival) ALB, ALT, AST, TBil, DBil, PT, INR, MELD score, symptoms, SE,	Adverse event: (-) 4wk: ALB, ALT, AST, Tbil, DBil, PT, INR, MELD score–improvement T > C (*P* < 0.05) 24 wk: ALB, PT, INR improvement T > C (*P* < 0.05), CSR: T= 54.5%, C = 26.5% (*P* = 0.015)

29	C1: FAP/BT 200 mL C2: FAP/BT 200 mL + PE T1: UCB 200 mL T2: UCB 200 mL+ PE FAP/BT/UCB: 1-3 x/wk, 2-4 wk PE 1500-3000 mL 1-3 x/wk, total 2-5x	C1: IV C2: IV T1: IV T2: IV	Severe viral hepatitis, severe hepatic dysfunction/(-)	RCT C1 = 39 (36 M) C2 = 45 (43 M) T1 = 38 (37 M) T2 = 31 (31 M)	BL-follow-up time not specified: SE, ALB, ALT, TBil, PTA, CD4, CD8, active T lymphocytes, IL2 SE (not mentioned in method)	SE–rash: C1 = 3, C2 = 10, T2 = 8, fever: C2 = 4 After treatment: ALB↑, PTA ↑: T2 > T1 > C2 > C1 TBil ↓: T2 > T1 > C2 > C1 CD4↑, active T lymphocytes↑, IL2 ↑: T2 > T1

T = treatment, BM-MSCs = bone marrow mesenchymal stem cells, P3 = passage-3, IV = intra venous, G-CSF = granulocyte colony stimulating factor, SC- subcutaneous, d = day/days, BW = body weight, ND = no data, C = control, MNC = mononuclear cells,HSA = human serum albumin,QD = four times a day, PB = peripheral blood,CD34SC = CD34 stem cells, auto = autologous, CT = computed tomography, wk = week/weeks, hu UC MSC = human umbilical cord mesenchymal stem cells, FAP = fresh adult plasma, BT = blood transfusion, PE = plasma exchange, UCB = umbilical cord blood, standard/supportive medical treatment-1 = SMT-1 = reduced glutathione, glycyrrhizin, ademetionine, polyenephosphatidylcholine, alprostadil, HSA, SMT-2 = close monitoring of emergency cases, if necessary: IV fluids, supplement of nutrition, zinc (for appetite), vitamin D (for osteoporosis), regular exercise to maintain muscle mass, management of pruritus, ascites, and portal hypertension, and avoidance of liver metabolized medications. IP = intraportal, BL = baseline, SMT-3 = vitamin B, calorie intake stimulation, alcohol abstinence, 4 wk prednisone 40 mg/day for severe ALD (Maddrey's score ≥ 32), HLC = hepatocyte like cells,SMT-4 = HSA, fresh plasma, vitamin K, according to patients' needs,SMT-5 = anti-HBV nucleoside analogue, liver protection, jaundice treatment, diuretic, SMT-6 = nutritional supplementation, HSA-10 g/day until s-Alb = 35 g/L, fresh frozen plasma (200-400 mL/day until INR = <1.5, entecavir (0.5 mg/day), S-adenosylmethionine (1.0 g/day), + treatment of complications, SMT-7 = lamivudine (100 mg daily), patients with ascites: diuretics (40 mg spironolactone +20 mg furosemide) daily, SMT-8= liver protection, liver enzyme activity and jaundice reduction, anti-HBV virus , treatment of complications, SMT-9= infusion of reduced glutathione, glycyrrhizin, ademetionine, polyenephosphatidylcholine, HSA. ACLF = acute on chronic liver failure, MELD = model of end-stage liver disease, SA = survival analysis, CTP = Child-Turcotte-Pugh, NS = no significant difference, HBVr = hepatitis C virus related, LT = liver transplantation, PEG-IFN = PEGylated Interferon *α*. P-no = participant number, M = male, OLPC = open labelled parallel controlled trial, ph = phase, RCT = randomized controlled trial. ALT = serum alanine aminotransferase, TBIL = total bilirubin, ALB = albumin, CHE = cholinesterase, PTA = prothrombin activity, PlC = platelet count,AST = aspartate aminotransferase, ICG-R15 = indocyanin green retension after 15 minutes, LL = lower limb, s- = serum-, Bil = bilirubin, PT = prothrombin time, PC = prothrombin concentration, INR = International normalized ratio, bU = blood urea, Cr = creatinine, F-BG = fasting - blood glucose, BGAM = blood glucose 2 hours after meal,pcoll III = procollagen III, GGT = gamma glutamyl transferase, DBil = direct bilirubin, SGOT = serum glutamic oxaloacetic transaminase = AST, SGPT = serum glutamic pyruvic transaminase = ALT, BUN = blood urea nitrogen, WBC = white blood count, NE = not evaluated, Hb = haemoglobin, HCC = hepatocellular carcinoma, LF = liver failure, MR = mortality rate, HR-QOL = health-related quality of life, CBC = complete blood count, ALP = alkaline phosphatase, HoS = hospital stay,S-RS = self-report symptoms (reduced appetite, abdominal distension, fatique),Enc = encephalopathy, Jau = jaundice, Hem/Mel = hematemesis/melena, Ed = edema, Asc = ascites, Eryt = erythema, Tremors=, Itch = itching, IBil = indirect Bil, AFP = *α* fetoprotein, imp = improvement, HRS = hepatorenal syndrome, HPC = hepatic progenitor cells, CEA = carcinoembryonic antigen, TG = triglycerides, TChol = total cholesterol. AE = cell therapy-related adverse event, SA = survival analysis, SE = side effect, h = hour/hours, AscR = ascites resolved, AcsD = ascites decrease, no = number of-, FDI = fever due to infection–resolved by treatment, CSR = cumulative survival rate, 1y- = 1 year-, NSD = no significant difference, SD = significant difference.

## Data Availability

All data were taken from the reference list.
